# Ambipolar solution-processed hybrid perovskite phototransistors

**DOI:** 10.1038/ncomms9238

**Published:** 2015-09-08

**Authors:** Feng Li, Chun Ma, Hong Wang, Weijin Hu, Weili Yu, Arif D. Sheikh, Tom Wu

**Affiliations:** 1 Department of Materials Science and Engineering, Division of Physical Sciences and Engineering, King Abdullah University of Science and Technology, Thuwal 23955-6900, Kingdom of Saudi Arabia

## Abstract

Organolead halide perovskites have attracted substantial attention because of their excellent physical properties, which enable them to serve as the active material in emerging hybrid solid-state solar cells. Here we investigate the phototransistors based on hybrid perovskite films and provide direct evidence for their superior carrier transport property with ambipolar characteristics. The field-effect mobilities for triiodide perovskites at room temperature are measured as 0.18 (0.17) cm^2 ^V^−1 ^s^−1^ for holes (electrons), which increase to 1.24 (1.01) cm^2 ^V^−1 ^s^−1^ for mixed-halide perovskites. The photoresponsivity of our hybrid perovskite devices reaches 320 A W^−1^, which is among the largest values reported for phototransistors. Importantly, the phototransistors exhibit an ultrafast photoresponse speed of less than 10 μs. The solution-based process and excellent device performance strongly underscore hybrid perovskites as promising material candidates for photoelectronic applications.

## Abstract (web summary)

Organolead halide perovskites exhibit good optoelectronic properties. Here, the authors fabricated and characterized ambipolar phototransistors based on solution-processed organolead triiodide and mixed-halide perovskites exhibiting high electron and hole mobilities and high photoresponsivities.

## Graphical abstract

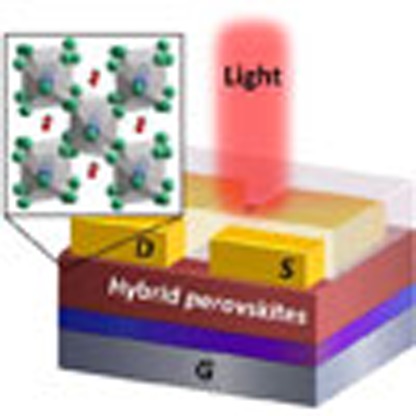

Methylammonium lead halide (CH_3_NH_3_PbX_3_, X=halogen) perovskites have been intensively pursued as light-harvesting materials in high-performance hybrid solid-state photovoltaic devices^1^,^2^,^3^,^4^,^5^,^6^,^7^,^8^,^9^,^10^,^11^,^12^,^13^. This class of materials was first discovered by Weber nearly 36 years ago^14^, and Mitzi and co-workers further revealed that halide perovskites combine the favourable carrier transport of inorganic semiconductors with the facile processing of organic materials^15^. In their pioneering work on field-effect transistors using hybrid perovskite (C_6_H_5_C_2_H_4_NH_3_)_2_SnI_4_ as channels, a high on/off ratio of 10^4^ and a hole mobility of 0.6 cm^2 ^V^−1 ^s^−1^ were reported^16^. The recent successes of halide perovskites in photovoltaic technologies can be primarily ascribed to their suitable, direct bandgap with large absorption coefficients and low-cost solution-based processing, as well as their excellent transport properties^17^,^18^,^19^,^20^. Long electron–hole diffusion lengths and carrier lifetimes have also been observed in perovskite films, indicating low recombination rates of charge carriers^21^,^22^. The properties of these emerging materials also led to photoelectronic applications, such as electrically pumped lasers, light-emitting diode/transistors and photodetectors^23^,^24^,^25^,^26^,^27^,^28^,^29^,^30^,^31^. Supplementary Table 1 summarizes the recent progresses on developing perovskite-based photodetectors. These hybrid perovskites can also be envisioned as good candidates for phototransistor, in which the gate bias, in addition to the incident light, is used as an additional parameter to modulate the channel transport^32^,^33^. Various types of semiconductors have been investigated as channel materials in phototransistors, such as Si, III–V semiconductors, ZnO, carbon nanotubes, quantum dots, organics and two-dimensional materials^34^,^35^,^36^,^37^,^38^,^39^,^40^,^41^,^42^,^43^,^44^,^45^,^46^,^47^,^48^,^49^. To date, there have been some pioneering lines of work on utilizing CH_3_NH_3_PbX_3_ as the active component in phototransistor-type devices^27^,^29^,^30^. However, the performance of these devices, particularly channel mobility and gate tuning, needs improvements.

In this work, we report on the fabrication and characterization of phototransistors on the basis of solution-processed organolead triiodide (CH_3_NH_3_PbI_3_) and mixed-halide (CH_3_NH_3_PbI_3−*x*_Cl_*x*_) perovskite films. The effects of gate voltage and light illumination on the transport of the perovskite channels were investigated. We found that these phototransistors exhibit clear ambipolar carrier transport characteristics, that is, they work in both accumulation (p-type) and inversion (n-type) modes. Highly balanced photo-induced carrier mobilities of 0.18 cm^2 ^V^−1 ^s^−1^ (holes) and 0.17 cm^2 ^V^−1 ^s^−1^ (electrons) were observed for CH_3_NH_3_PbI_3_, which increase to 1.24 cm^2 ^V^−1 ^s^−1^ (holes) and 1.01 cm^2 ^V^−1 ^s^−1^ (electrons) for the doped variant CH_3_NH_3_PbI_3−*x*_Cl_*x*_. As an important figure-of-merit for phototransistors, the photoresponsivity (*R*) of our perovskite phototransistor reaches 320 A W^−1^. Furthermore, we observed that the phototransistors exhibit an ultrafast photoresponse time of less than 10 μs. The results indicate that solution-processed hybrid perovskites are very promising materials for constructing high-performance phototransistors, and they warrant exploration for use in other photoelectronic applications.

## Results

### Hybrid perovskite phototransistor

**Figure 1:**
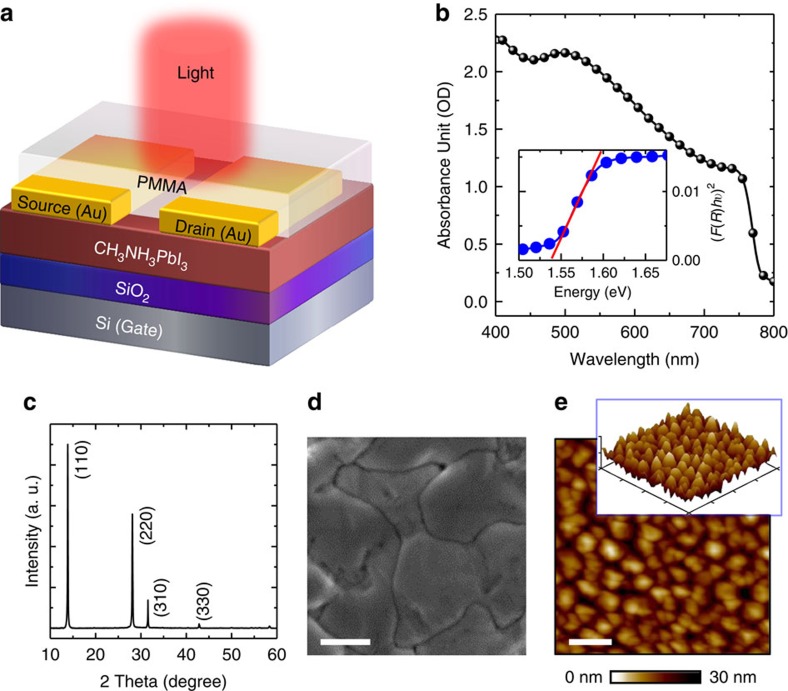
Hybrid perovskite phototransistors. (**a**) Schematic of the phototransistor with a channel of hybrid perovskite CH_3_NH_3_PbI_3_. (**b**) Ultraviolet–visible absorption of the 100-nm CH_3_NH_3_PbI_3_ film. The inset shows a direct bandgap of ∼1.53 eV. (**c**) X-ray diffraction spectrum of the CH_3_NH_3_PbI_3_ film. (**d**) Top-view SEM image of the perovskite film. Scale bar, 0.5 μm. (**e**) Tapping-mode AFM height image of the perovskite film. Scale bar, 1.0 μm. Inset: the corresponding three-dimensional topographic image.

A schematic device structure of a bottom-gate, top-contact hybrid perovskite CH_3_NH_3_PbI_3_-based phototransistor is illustrated in Fig. 1a. A heavily n-doped Si wafer with a 300-nm SiO_2_ surface layer (capacitance (*C*_i_) of 15 nF cm^−2^) was employed as the substrate. The perovskite films were grown using the two-step vapour-assisted solution process^11^, and the experimental details are given in the Methods section. In our devices, we coated poly(methyl methacrylate) (PMMA) as the protective layer on top of the perovskite channel to prevent the diffusion of moisture and/or atmospheric oxygen^21^. In optimizing phototransistor devices, the semiconducting channel thickness is a key parameter. On the one hand, if the semiconducting channel is too thin, it will not absorb sufficient light^10^. In addition, pinholes in the thin perovskite films will cause inhomogeneous conduction in the channel. On the other hand, if the film is too thick, light from the top may not be able to penetrate the whole film, and the bottom gate will not effectively modulate the channel. In our work, the thickness of the CH_3_NH_3_PbI_3_ film prepared in the phototransistors is optimized at 100 nm, as characterized using atomic force microscopy (AFM)^50^. The detailed AFM measurement results are shown in Supplementary Fig. 1.

Figure 1b shows the light absorption data of the CH_3_NH_3_PbI_3_ film. For this measurement, the thin films were prepared on a glass substrate using the same processing parameters as those used in fabricating the phototransistors. The strong and broad absorption in the ultraviolet and visible-light range, particularly from 400 to 760 nm, reveals that the perovskite layer is a good light absorber. Furthermore, the absorption edge for the perovskite film is very sharp, suggesting a direct bandgap nature^7^,^18^. Overall, the direct bandgap of 1.53 eV and the favourable light absorption properties make perovskite CH_3_NH_3_PbI_3_ films very promising materials for constructing high-performance phototransistors.

### Characterizations of the perovskite thin films

To investigate the crystallinity and microstructure of the perovskite thin films, X-ray diffraction (XRD) measurements were carried out. Figure 1c shows the X-ray diffraction pattern of the as-prepared CH_3_NH_3_PbI_3_ film on a SiO_2_/Si substrate. Strong peaks at 14.08°, 28.41°, 31.85° and 43.19° can be assigned to (110), (220), (310) and (330) diffractions of CH_3_NH_3_PbI_3_, respectively, indicating that the halide perovskite films possess the expected orthorhombic crystal structure with high crystallinity^17^. Notably, the absence of a diffraction peak at 12.65° suggests that the level of the PbI_2_ impurity phase is negligible^11^. The surfaces of the perovskite CH_3_NH_3_PbI_3_ films were further evaluated via scanning electron microscopy (SEM) and AFM measurements. Figure 1d shows the surface morphology of the as-grown perovskite thin film grown on the SiO_2_/Si substrate. The perovskite film appears smooth without pinholes, and the uniform grains have sizes up to hundreds of nanometres. These structural characters are promising for achieving high performance in phototransistors. As shown in Fig. 1e, the film surface was also characterized using AFM, and the root-mean-square roughness is ∼10.5 nm in a typical scanning area of 5.0 μm × 5.0 μm. The three-dimensional AFM image in the inset of Fig. 1e further demonstrates the smooth surface of the perovskite film.

### Phototransistor characterization

**Figure 2:**
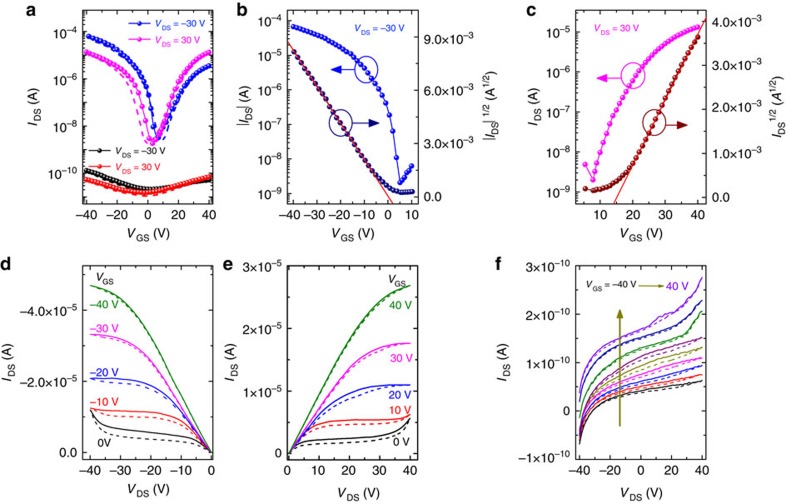
Ambipolar characteristics of the hybrid perovskite phototransistors. (**a**) Transfer characteristics of a perovskite-based phototransistor in the dark (black and red symbols) and under light illumination (blue and magenta symbols). (**b**,**c**) Representation of the transfer characteristics of p-channel and n-channel behaviours, respectively, under light illumination. (**d**,**e**) Respective output properties of the device under light condition. (**f**) Output curves of the phototransistor in the dark condition. Solid and dashed curves were measured during forward and backward sweepings, respectively.

Figure 2a shows a representative set of the transfer characteristics, which are the drain current versus gate voltage (*I*_DS_−*V*_GS_) data, of a bottom-gate top-contact CH_3_NH_3_PbI_3_ phototransistor. The devices were measured both in the dark and under white-light illumination (power density: 10 mW cm^−2^) at the drain voltages (*V*_DS_) of −30 and 30 V. An ambipolar high performance can be clearly observed in the transfer characteristics under light illumination, and the V shape of the transfer curves is similar to those of previous reports on ambipolar transistors^51^,^52^,^53^,^54^,^55^,^56^. The current of the illuminated channel can reach 0.1 mA. In contrast, for the transfer characteristics measured under the dark condition, *I*_DS_, remains below 0.5 nA.

Figure 2b,c shows the plots of *I*_DS_^1/2^ and *I*_DS_ as functions of *V*_GS_ for the perovskite phototransistor measured under light illumination. The plots appear linear for a large range of *V*_GS_ for both p- and n-type transports. The field-effect mobility (*μ*) and threshold voltage (*V*_TH_) can be extracted using the saturated *I*_DS_ versus *V*_GS_ relationship^52^,^53^:


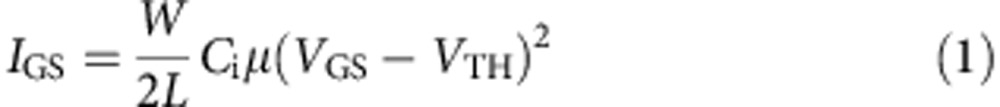


where *W*, *L* and *C*_i_ are the channel width, the channel length and the gate capacitance per unit area, respectively. Accordingly, the photo-induced hole and electron mobilities are derived as 0.18 and 0.17 cm^2 ^V^−1 ^s^−1^, respectively, in the saturation region. Thus, it is clear that under light illumination the perovskite-based phototransistor exhibits ambipolar behaviour with balanced hole and electron mobilities.

Under light illumination, the output characteristics (that is, *I*_DS_ versus *V*_DS_ data at different *V*_GS_) obtained for the device operating in the hole-enhancement and electron-enhancement modes are displayed in Fig. 2d,e, respectively. At low *V*_GS_, the device exhibits ambipolar transport with diode-like current–voltage (*I–V*) characteristics; however, at high *V*_GS_, unipolar transport with standard linear-to-saturation *I*–*V* transistor characteristics was observed. Figure 2f shows the output characteristics of the phototransistor measured in the dark. It can be clearly seen that there is no obvious field effect, and *I*_DS_ remains at the level of 10^−10 ^A, which is consistent with the transfer characteristics.

For measurements performed in the dark, as shown in Fig. 2a, *I*_DS_ remains less than 0.5 nA when *V*_GS_ increases from −40 to +40 V, and the obtained on/off ratio is less than 10. In contrast, regarding device performance under light illumination, the on current is significantly enhanced to 10^−4 ^A, while the off current also increases to 2.0 × 10^−9 ^A. Furthermore, the on/off ratios under illumination are boosted to 3.32 × 10^4^ for p-type and 8.76 × 10^3^ for n-type transports. The significant gate-tuning effect on illumination with respect to the dark measurements implies that the photo-excited carriers dominate the channel transport; thus, the device has potential for use in other photoelectronic applications such as photosensors.

Furthermore, it should be noted that both the transfer curves and the output curves exhibit weak but notable hysteresis effects (Fig. 2), which were also reported for solar cells, memristors and light-emitting field-effect transistors on the basis of hybrid perovskites^31^,^57^,^58^,^59^. One origin of the hysteresis effects was speculated to be the screening effects arisen from the field-induced drift of methylammonium cations^59^. In addition, charge traps and surface dipoles at the untreated semiconductor–dielectric interface may also attribute to the hysteresis effect^53^. In Fig. 2, the hysteresis effect in the phototransistor operation is not substantial, which is presumably a result of the high quality of the perovskite films.

To examine the effect of the device structure, we also fabricated a bottom-gate bottom-contact perovskite phototransistor (schematic shown in Supplementary Fig. 2). Supplementary Fig. 3 shows the ambipolar transfer characteristics of the devices under light illumination, which is similar to that of the bottom-gate top-contact devices. In this particular device, both the electron and hole mobilities were measured as 0.17 cm^2 ^V^−1 ^s^−1^, suggesting a very balanced ambipolar transport under light illumination. These results reveal that the ambipolar performance of phototransistors is an intrinsic property of CH_3_NH_3_PbI_3_ and is independent of the device architecture.

**Figure 3:**
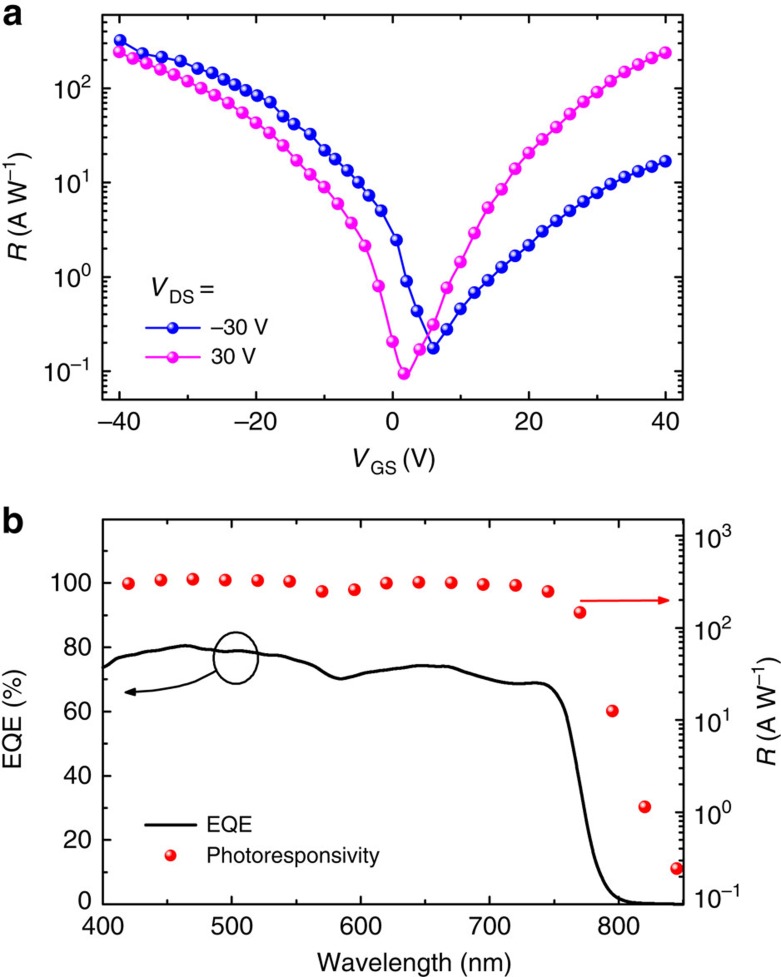
Performance of the perovskite phototransistors. (**a**) Photoresponsivity (*R*) characteristics of the CH_3_NH_3_PbI_3_ phototransistor under white-light illumination (power density= 10 mW cm^−2^). (**b**) EQE and *R* of the hybrid perovskite photodetector at different wavelength and a *V*_DS_ of −30 V.

Photoresponsivity (*R*) is the key parameter for evaluating the performance of phototransistors, and its value can be obtained using transfer characteristics. *R* is given by the following equation:


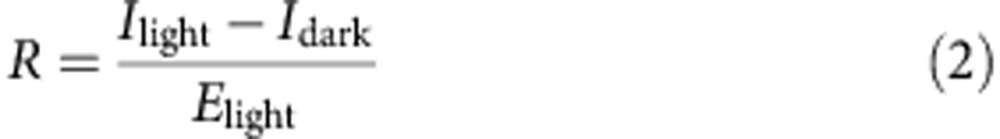


where *I*_light_ and *I*_dark_ stand for the drain currents under light illumination and in the dark, respectively, and *E*_light_ is the power of incident illumination. *R* as a function of *V*_GS_ is shown in Fig. 3a. The maximum *R* value of 320 A W^−1^ in the On state (*V*_GS_=−40 V) was obtained at a power density of 10 mW cm^−2^. We also examined the phototransistors with various perovskite film thicknesses (shown in Supplementary Fig. 4), and we found that the performance of devices with the active layer thickness of ∼100 nm is optimal. Note that this parameter achieved in perovskite phototransistors is substantially higher than those of most other reported functional materials^38^,^40^,^42^,^43^,^44^,^45^,^47^,^48^. For example, phototransistors based on organic semiconductors and hybrid materials were reported with *R* values typically below 1.0 A W^−1^ (ref. 45). This superb performance of hybrid perovskites in phototransistors can be attributed to their excellent light absorption properties, extremely low dark current and high photocurrent.

The external quantum efficiency (EQE) and photoresponsivity (*R*) as the function of wavelength in our phototransistor are shown in Fig. 3b. The device exhibits a broad photoresponse range from 400 to 800 nm, and its maximum EQE is approximately 80%. Moreover, the device shows high *R* values in the wavelength range of 400–750 nm, and hence it is suitable for visible-light broadband photodetection. In addition, we found that the spectral sensitivities of the phototransistor are mainly determined by the absorption spectra of the perovskite film (Fig. 1b).

### CH_3_NH_3_PbI_3−*x*_Cl_*x*_-based phototransistors

**Figure 4:**
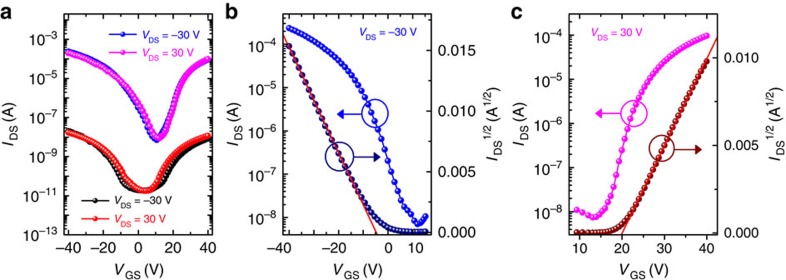
Characteristics of the CH_3_NH_3_PbI_3−*x*_Cl_*x*_-based phototransistors. (**a**) Transfer curves of the CH_3_NH_3_PbI_3−*x*_Cl_*x*_ phototransistor in the dark (black and red symbols) and under light illumination (blue and magenta symbols). (**b**,**c**) Representation of the transfer characteristics of p-type and n-type behaviours, respectively, under light illumination.

It was reported that Cl might act as a crystallization-retarding and -directing agent^60^, which benefits the growth of perovskite domains and thus improves the transport properties of the perovskite films^21^,^22^. Therefore, we fabricated mixed-halide perovskite CH_3_NH_3_PbI_3−*x*_Cl_*x*_-based phototransistors. Indeed, we found that the presence of Cl improves the surface smoothness of the perovskite films (Supplementary Fig. 5). The transfer characteristics of the CH_3_NH_3_PbI_3−*x*_Cl_*x*_-based phototransistor in dark and light conditions are shown in Fig. 4a. Under light illumination (10 mW cm^−2^), the device shows clear ambipolar behaviour, similar to that of CH_3_NH_3_PbI_3_-based devices. As shown in Fig. 4b,c, the slopes of *I*_DS_^1/2^ versus *V*_GS_ are linear for a large range of *V*_GS_ for both p-type and n-type transports, which demonstrates the high quality of our phototransistors. On the basis of equation (1), the obtained mobilities of photo-induced holes and electrons are 1.24 and 1.01 cm^2 ^V^−1 ^s^−1^, respectively. However, unlike the case of CH_3_NH_3_PbI_3_ phototransistors, *I*_DS_ in CH_3_NH_3_PbI_3−*x*_Cl_*x*_ devices can reach 10 nA under the dark condition, and the hole and electron mobilities are 1.62 × 10^−4^ and 1.17 × 10^−4 ^cm^2 ^V^−1 ^s^−1^, respectively (Supplementary Fig. 6). These results obtained under the dark condition imply that the CH_3_NH_3_PbI_3−*x*_Cl_*x*_ channels have substantially higher conductivity and mobility than the CH_3_NH_3_PbI_3_ ones. As shown in Supplementary Fig. 7, the maximum *R* value of the CH_3_NH_3_PbI_3−*x*_Cl_*x*_ phototransistor is ∼47 A W^−1^ at *V*_GS_=−40 V. The lower *R* of the CH_3_NH_3_PbI_3−*x*_Cl_*x*_ phototransistor compared with that of the CH_3_NH_3_PbI_3_ phototransistor is primarily because of its higher dark current. Nevertheless, this *R* value is still comparable to the highest ones reported for other functional materials^38^,^40^,^42^,^43^,^44^,^45^,^47^,^48^.

### Characterization of photocurrent response

**Figure 5:**
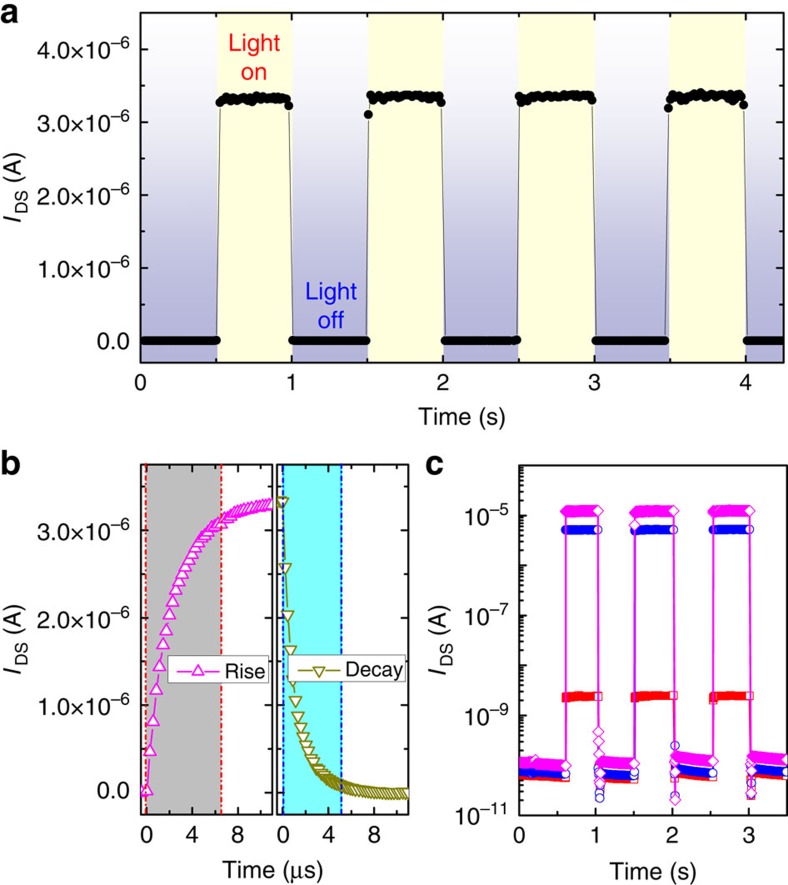
Photocurrent response of the perovskite phototransistors. (**a**) Photocurrent responses of the phototransistors on light illumination showing time-dependent photosensitivity with a time interval of 0.5 s at *V*_GS_=−30 V and *V*_DS_=−30 V. (**b**) Temporal photocurrent responses highlighting a rise time of 6.5 μs and a decay time of 5.0 μs. (**c**) Gate voltage-dependent photoresponses measured at *V*_DS_=−30 V. The device was measured at *V*_GS_ of 0 V (red squares), −30 V (blue circles) and −40 V (magenta diamonds).

Fast response to optical signals, which is a result of efficient charge transport and collection, is critical for optoelectronic devices. The time-dependent photocurrent was recorded when the white light (10 mW cm^−2^) was turned on and off regularly. Figure 5a shows the response of photocurrent to optical pulses at a time interval of 0.5 s when the device is biased at *V*_DS_=−30 V and *V*_GS_=−30 V. We found that the dynamic photoresponse of the perovskite phototransistors was stable and reproducible. The photocurrent quickly increases as soon as the light is turned on and then drops to the original value when the light is turned off, indicating that the device functions as a good light-activated switch.

Figure 5b shows the temporal photocurrent response of the perovskite phototransistor. The measured switching times for the rise (*I*_DS_ increasing from 0 to 80% of the peak value) and decay (*I*_DS_ decreasing from the peak value to 10%) of the photocurrent are ∼6.5 and 5.0 μs, respectively, indicating an ultrafast response speed. The decay time of the photodetector has previously been estimated as the carrier recombination time (lifetime, *τ*_life_)^39^,^41^,^43^. Recently, using a solar-cell-type photodetector, Dou *et al.* obtained response times below 200 ns (ref. 26). In fact, photo-induced electron–hole pairs in the pristine perovskite recombine within a few picoseconds (that is, within the lifetime of the photo-excited electrons)^30^. However, in the presence of positive (negative) gate voltages, the photo-generated electrons (holes) are accumulated in the channel and acquire a higher mobility, while the other type of charges remain trapped in the perovskite channel. Thus, the recombination of the photo-excited electron–hole pairs in the perovskite channel is reduced, resulting in increased carrier lifetime (*τ*_life_)^43^,^61^. Nevertheless, the temporal photocurrent responses of our perovskite phototransistors are still substantially faster than those of most organic, quantum dot and hybrid photodetectors (typically in the order of milliseconds)^36^,^41^,^42^,^43^,^44^,^45^,^46^,^47^,^48^,^49^.

Figure 5c shows the characteristics of photo-switched channel current at *V*_DS_=−30 V and various *V*_GS_. As shown, on light illumination, a larger *V*_GS_ can induce a more significant increase in photocurrent. In particular, the photocurrent is ∼2.76 × 10^−9 ^A at *V*_GS_=0 V, whereas it dramatically increases to 1.21 × 10^−5 ^A at *V*_GS_=−40 V. These results demonstrate that the switching of the photocurrent could be tailored to a large degree using the gate voltages, enabling highly tunable photodetection.

### Device stability

**Figure 6:**
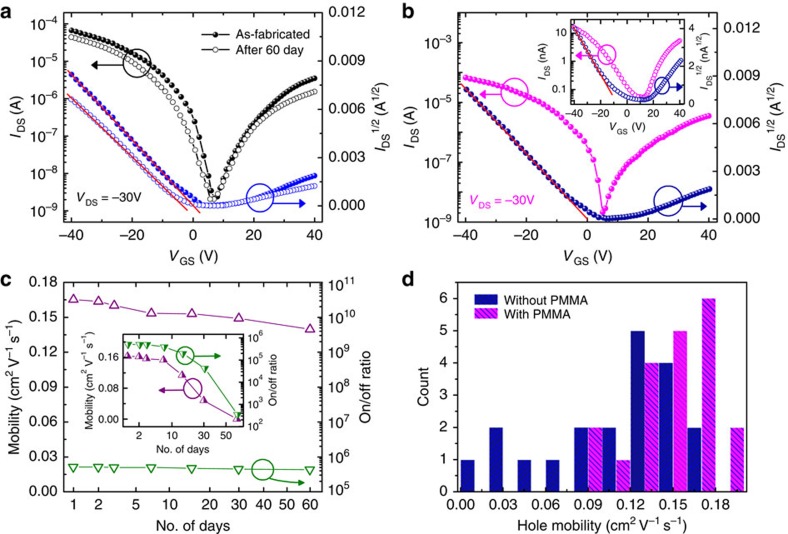
Stability and reproducibility of the perovskite phototransistors. (**a**) Transfer curves under light illumination of the Device-P (with the PMMA-protective layer) immediately following fabrication and after storing under ambient conditions for 60 days. (**b**) Transfer characteristics under light illumination of the as-fabricated Device-U (without PMMA-protective layer). Inset, data measured after storing under ambient conditions for 60 days. (**c**) Mobility and on/off ratio of Device-P measured during 60 days of storage under ambient conditions. Stability data of Device-U are also shown in the inset for comparison. (**d**) Histogram of the hole mobilities measured on 20 devices with the PMMA-protective layer and 20 devices without the protective layer.

To enhance the stability of perovskite phototransistors, we coated some bottom-gate top-contact devices with PMMA-protective layers (noted as Device-P). These devices were stored under ambient conditions without isolation from exposures to atmospheric air and moisture. Previous lines of work on perovskite solar cells have demonstrated that coating the PMMA-protective layer is an effective strategy to improve the ambient stability of perovskites by forming kinetic barriers against the diffusion of moisture and/or atmospheric oxygen^21^. Under light illumination, the transfer curves of a device immediately following fabrication and after storing under ambient conditions for 60 days are essentially the same, except slight decreases in the channel current (shown in Fig. 6a), revealing excellent device stability. In contrast, the unprotected devices without the PMMA layer (noted as Device-U) exhibited sustantially weaker stability. Figure 6b shows the transfer characteristics of a newly fabricated Device-U, which are similar to those of Device-P. The inset of Fig. 6b shows the transfer curve of Device-U that had been stored under ambient conditions for 60 days. Notably, the device showed *I*_DS_ of only ∼10^−8^A at *V*_GS_=−40 V, which is nearly 10^4^-fold lower than the *I*_DS_ of the newly fabricated Device-U. Figure 6c shows the mobility and on/off ratio of the Device-P measured at different intervals over a period of 60 days. No apparent statistical variations in the mobilities or on/off ratio were observed, confirming the excellent stability of the encapsulated phototransistors. For the Device-U (shown in the inset of Fig. 6c), both the mobility and the on/off ratio declined continuously during 60 days of storage. However, it should be mentioned that the performance of Device-U changed very little over the first 7 days, indicating that unprotected devices must be measured quickly to achieve reproducible data.

High reproducibility and stability are critical for photoelectronic devices and their integrations in real-world applications. Figure 6d shows the distributions of hole mobility for both PMMA-protected and -unprotected as-fabricated devices. More than 70% of the Device-P group (20 devices) show hole mobility ranging from 0.13 to 0.19 cm^2 ^V^−1 ^s^−1^. However, among the 20 devices in the Device-U group, there are notable fluctuations of mobility values: some are similar to Device-P, while others are even below 9 × 10^−2 ^cm^2 ^V^−1 ^s^−1^. The perovskite phototransistors without the PMMA layer often exhibit low reproducibility, primarily because of their high sensitivity towards moisture, room light and other factors under ambient conditions. Thus, coating PMMA-protective layers should be considered as a general protocol for enhancing device stability and reproducibility.

## Discussion

In this work, ambipolar phototransistors based on solution-processed hybrid perovskites were fabricated and characterized. Owing to the superior optical and electronic properties of hybrid perovskites, our solution-processed phototransistors demonstrated high performances and tunability. Under light illumination, these devices exhibit reliable high performance with balanced carrier mobilities and on/off ratios of ∼10^4^ for both p- and n-type transports. Remarkably, the phototransistors show excellent figures-of-merit such as excellent photoresponsivity (320 A W^−1^) and ultrafast response speed (less than 10 μs).

In such phototransistors, both light illumination and gate bias can be used to modulate the transport of semiconducting perovskite channels. Through the capacitive coupling, the gate bias is expected to effectively separate the photo-generated holes and electrons, increasing their recombination time, equivalently *τ*_life_ (ref. 43, 61). The photoconductive gain (*G*), that is, the number of carriers collected per photo-induced carrier, is given by the equation:


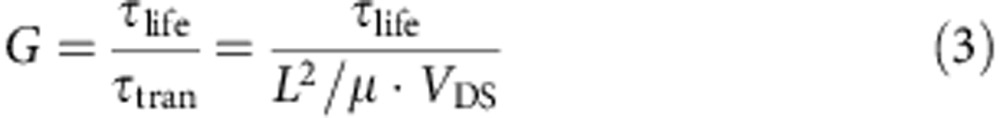


where *τ*_tran_ is the carrier transit time^39^,^41^,^42^,^43^. For our devices with mobility in the range of 0.2–2.0 cm^2 ^V^−1 ^s^−1^, *G* is estimated to be ∼10–10^2^. Through biasing the gate terminal, our phototransistor architecture is advantageous for enhancing the photoresponse.

Furthermore, according to equation (3), the device performance should also benefit from high carrier mobility (*μ*). In our phototransistors, the field-effect hole/electron mobility was measured as 0.18/0.17 cm^2 ^V^−1 ^s^−1^ for CH_3_NH_3_PbI_3_ and 1.24/1.01 cm^2 ^V^−1 ^s^−1^ for CH_3_NH_3_PbI_3−*x*_Cl_*x*_, this is consistent with the Cl-enhanced electron–hole diffusion lengths observed in previous lines of work on perovskite solar cells^21^,^22^. However, better phototransistor performance was achieved in the undoped CH_3_NH_3_PbI_3_ channel as a result of suppressed dark current. We should note that, although the field-effect mobility measured in our perovskite phototransistors is impressive for solution-processed materials, there is still room for improvements. Tailoring the deposition procedures and developing appropriate doping strategies are promising to optimize the transport properties of perovskite channels^62^,^63^.

Furthermore, according to equation (3), reducing the channel length (*L*) is a straightforward way to improve the performance of phototransistors. In the present device structure, the channel length is 50 μm, which are much longer than the diffusion lengths of the carriers. Therefore, light-induced carriers are scattered many times by structural defects and grain boundaries before reaching the electrodes^64^,^65^. Much higher carrier mobility is expected in phototransistors with shorter channel lengths, which could further improve the phototransistor performance. Shrinking the channel dimensions may also help decrease the *V*_DS_ required for the device operation and reduce the energy consumption. Overall, our findings strongly support the use of hybrid perovskites as active materials in high-performance ambipolar phototransistors, and open new doors for employing such solution-processed perovskites in other photoelectronic applications.

## Methods

### Perovskite preparation and device fabrication

CH_3_NH_3_I was synthesized according to a previously reported procedure^2^,^6^,^11^. First, 24 ml of methylamine (33 wt% in absolute ethanol, Sigma) and 10 ml of hydroiodic acid (57 wt% in water, Aldrich) were mixed to react in a 250-ml round-bottomed flask at 0 °C for 2 h with stirring. The precipitate was recovered via evaporation at 50 °C for 1 h. The product, methylammonium iodide CH_3_NH_3_I, was washed with diethyl ether by stirring the solution for 30 min, which was repeated three times. The product was finally dried at 60 °C in a vacuum oven for 24 h. The perovskite films were prepared according to the vapour-assisted solution process^11^. The heavily n-doped Si wafers with 300-nm SiO_2_ surface layers (capacitance of 15 nF cm^−2^) were used as the substrates. They were successively cleaned with diluted detergent, rinsed with deionized water, acetone and ethanol and dried with dry nitrogen. After the oxygen plasma treatment, the solution of 0.3 M PbI_2_ or PbCl_2_ (Sigma) in dimethylformamide was spin-coated on the cleaned Si substrates at 5,000 r.p.m. for 40 s and dried at 110 °C for 15 min. CH_3_NH_3_I powders were spread around the PbI_2_- or PbCl_2_-coated substrates, and a Petri dish was placed over the samples. The substrates were heated at 150 °C for 8 h. After cooling, the as-prepared films were washed with isopropanol and dried at 65 °C for 5 min. Finally, Ti/Au (5 nm/80 nm) source (S) and drain (D) electrodes were deposited via thermal evaporation through a shadow mask, defining a channel length (*L*) of 50 μm and a channel width (*W*) of 1,000 μm. Furthermore, the fabricated devices were annealed in a glove box at 50–60 °C for 10 min to reduce the charge traps and improve the contact between the active layer and the S/D electrodes^66^. The bottom-gate bottom-contact devices were fabricated following similar procedures, except that Au S/D electrodes were deposited on the Si substrates before spin-coating the PbI_2_ or PbCl_2_ solution. To improve device stability, thin layers of PMMA (average molecular weight ∼350,000 by Gel Permeation Chromatography, Sigma-Aldrich) were spin-coated on the surfaces of the perovskite films at 8,000 r.p.m. for 60 s.

### Measurements and experimental set-up

Perovskite film thickness was assessed using the scratch method via AFM (Bruker Dimension ICON). X-ray diffraction patterns of the perovskite films were recorded using a Bruker D8 ADVANCE diffractometer with Cu *Kα* (*λ*=1.5406 Å) radiation. A field-emission SEM (FEI Nova Nano 630) was used to acquire surface SEM images. In addition, the morphology of the perovskite films was determined via AFM measurements in the tapping mode. The absorption spectra were measured using an Agilent Cary 6000i UV-Visible-NIR spectrometer. The devices *I–V* measurements were performed using a Keithley 4200 Semiconductor Parametric Analyzer and a Signotone Micromanipulator S-1160 probe station. A light-emitting diode (white light, 10 mW cm^−2^) attached to the microscope of the probe station was used as the light source. During the measurements, the samples were kept at room temperature in the ambient atmosphere. The EQE of the device was measured using an Oriel IQE-200 measurement system.

## 

## Additional information

**How to cite this article:** Li, F. *et al.* Ambipolar solution-processed hybrid perovskite phototransistors. *Nat. Commun.* 6:8238 doi: 10.1038/ncomms9238 (2015).

## Supplementary Material

Supplementary Information Supplementary Figures 1-7, Supplementary Table 1 and Supplementary References.
