# Supplementary material for: Ambipolar solution-processed hybrid perovskite phototransistors
Source: Nat Commun. 2015 Sep 8;6:8238. doi: 10.1038/ncomms9238 (PMC4569843; doi:10.1038/ncomms9238)
Supplement: Supplementary Information — Supplementary Figures 1-7, Supplementary Table 1 and Supplementary References. [file ncomms9238-s1.pdf]

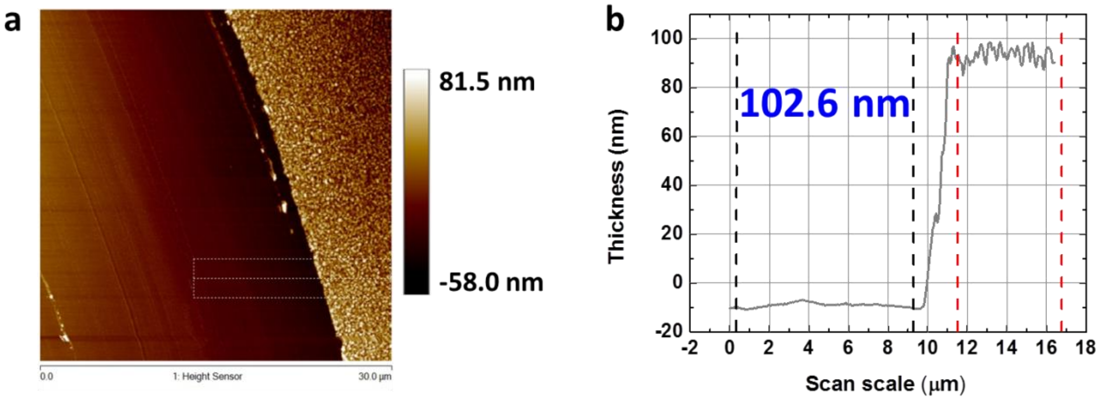

Supplementary Figure 1. Film thickness measurement. (**a**) AFM images of the perovskite film with a straight edge which was created by scratching with a tweezer. (**b**) Profile along the line highlighted in (**a**). This profile was used to estimate the thickness of the perovskite film.

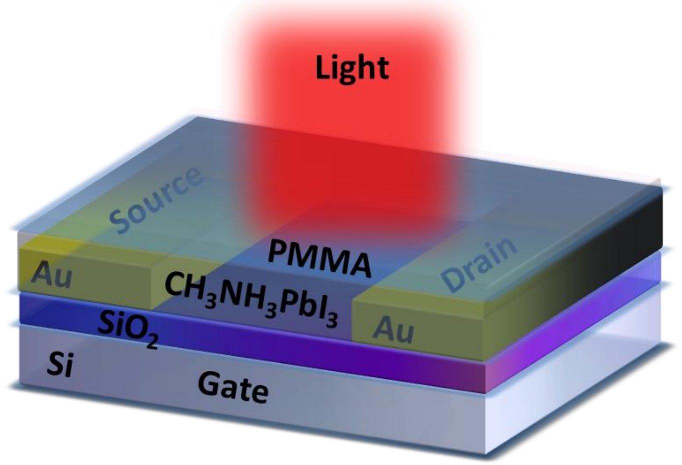

Supplementary Figure 2. Device schematic. Schematic diagram for a $CH_3NH_3PbI_3$-based phototransistor with the bottom-gate bottom-contact structure.

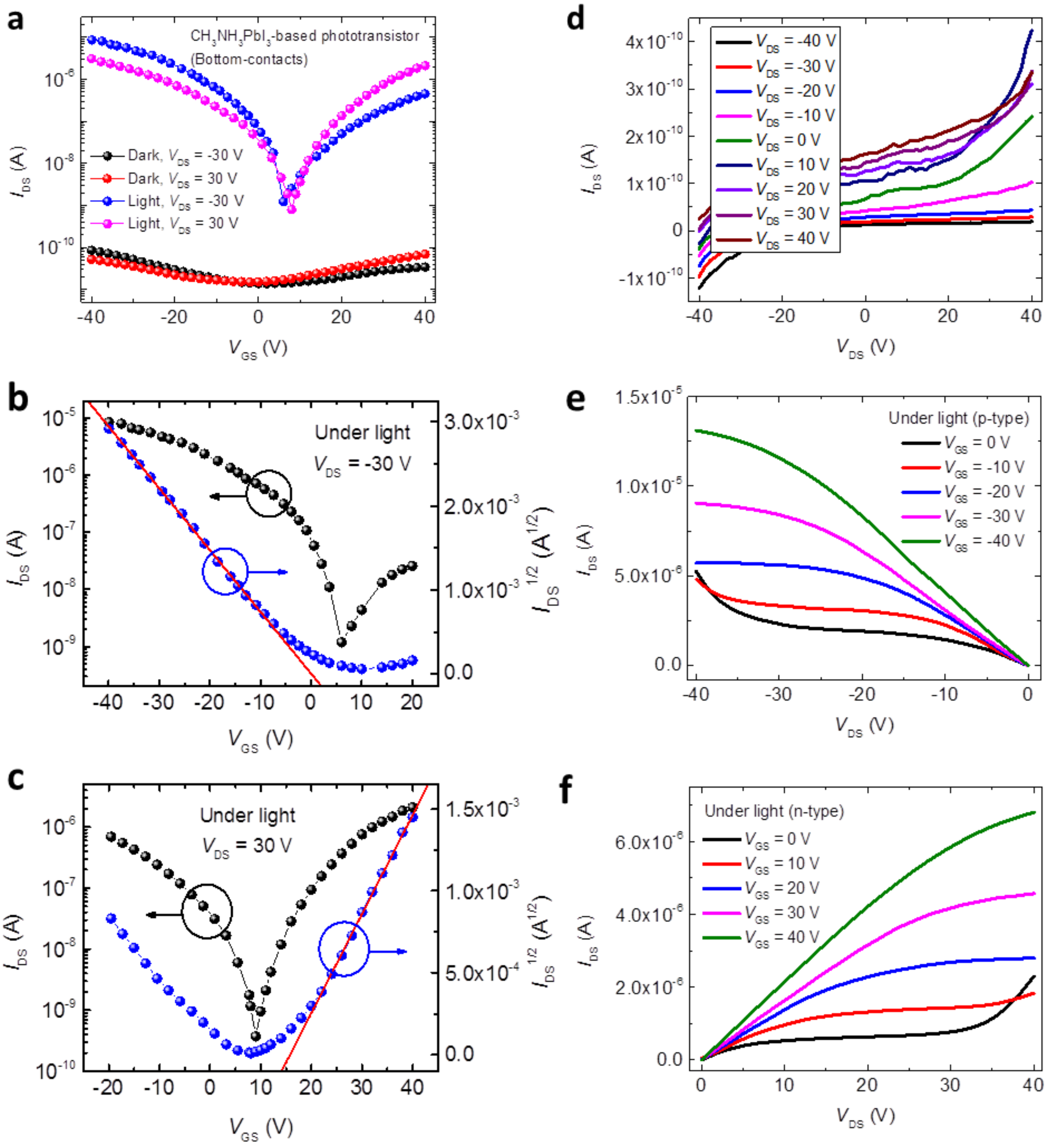

Supplementary Figure 3. Performance of the bottom-gate, bottom-contact perovskite phototransistors. (**a**) Transfer curves for the bottom-gate, bottom-contact phototransistor device. (**b**) and (**c**) represent, respectively, the transfer characteristics of p-type behavior and n-type behavior. (**d**), (**e**) and (**f**) are output curves of the phototransistor in dark and under light illumination.

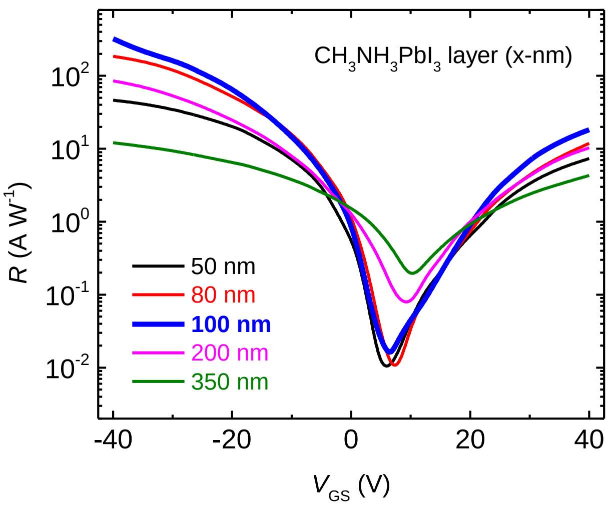

Supplementary Figure 4. Photoresponsivity of the devices with various film thicknesses. The device with a thickness of about 100 nm was found to present the optimal performance.

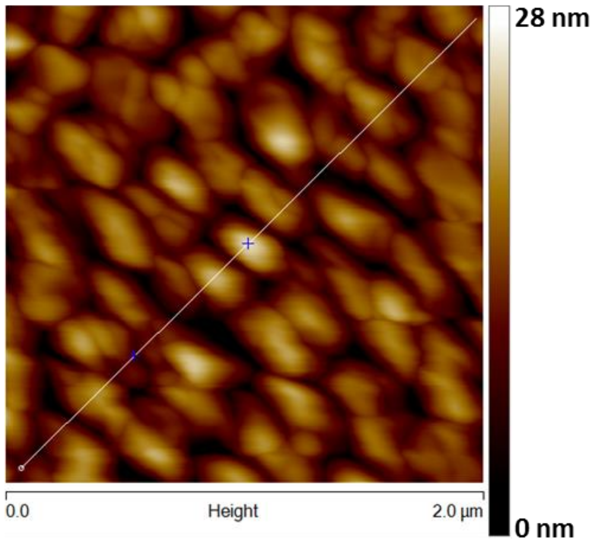

Supplementary Figure 5. Atomic force microscopy (AFM) characterization of the $CH_3NH_3PbI_{3-x}Cl_x$ film surface. The root-mean square roughness is approximately 8.95 nm, demonstrating a smoother surface compared to the $CH_3NH_3PbI_3$ film (shown in Fig. 1d in the main text).

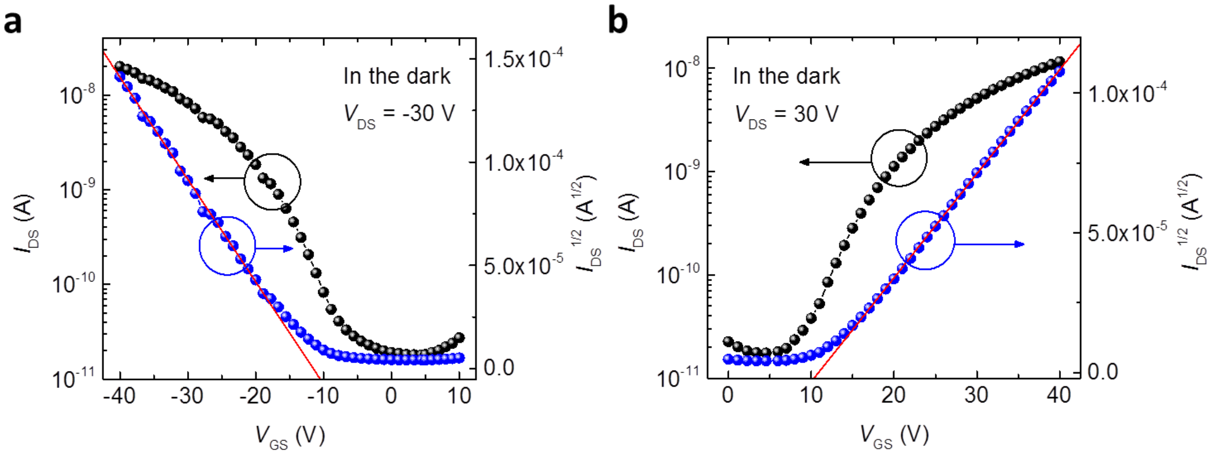

Supplementary Figure 6. Transfer curves of a hybrid perovskite $CH_3NH_3PbI_{3-x}Cl_x$-based phototransistor in the dark. (**a**) and (**b**) represent the transfer characteristics of a hybrid perovskite $CH_3NH_3PbI_{3-x}Cl_x$-based phototransistor in the p-type and n-type regimes, respectively.

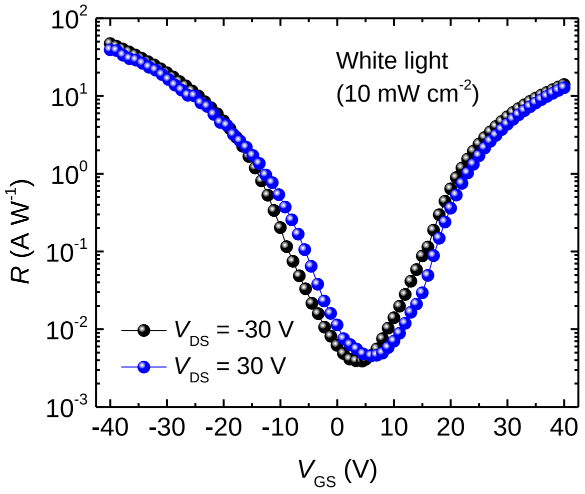

Supplementary Figure 7. Photoresponsivity (*R*) data. Photoresponsivity (*R*) measured on the perovskite $CH_3NH_3PbI_{3-x}Cl_x$-based phototransistor.

Supplementary Table

Supplementary Table 1. Performance of reported photodetectors based on hybrid perovskites.

Ref.	Year	Materials	Configuration	Responsivity ($A\ W^{-1}$)	Detectivity (Jones)	Response time
1	2014	$CH_3NH_3PbI_3$ film	solar cell	Photocurrent amplification > 100		
2	2014	$CH_3NH_3PbI_3/TiO_2$ film	photodetector	0.49×10^{-6}		0.02 s
3	2014	$CH_3NH_3PbI_3$ film	photodetector	3.49		< 0.2 s
4	2014	$CH_3NH_3PbI_{3-x}CI_x$ film	photodetector		$\sim10^{14}$	160 ns
5	2014	$CH_3NH_3PbI_3$ nanowires	phototransistor	5×10^{-3}		< 500 μs
6	2015	$CH_3NH_3PbI_3$ film	photodetector	14.5		0.2 μs
7	2015	Graphene- $CH_3NH_3PbI_3$ composites	phototransistor	180	$\sim10^{9}$	87 ms
8	2015	$CH_3NH_3PbI_3$ film	photodetector	242		5.7 ± 1.0 μs
9	2015	$CH_3NH_3PbI_3$ film	photodiode		3×10^{12}	< 5 μs
10	2015	$CH_3NH_3PbI_3$ film	photodetector		7.4×10^{12}	120 ns
11	2015	$CH_3NH_3PbI_3$ film	optocoupler	1.0		20 μs
12	2015	$CH_3NH_3PbI_3$ nanowires	photodetector	1.3	2.5×10^{12}	0.3 ms

Supplementary References

1. Moehl, T. *et al.* Strong photocurrent amplification in perovskite solar cells with a porous TiO2 blocking layer under reverse bias. *J. Phys. Chem. Lett.* **5**, 3931-3936 (2014).
2. Xia, H.-R. *et al*. Organohalide lead perovskite based photodetectors with much enhanced performance. *Chem. Commun*. **50**, 13695–13697 (2014).
3. Hu, X. *et al.* High-Performance Flexible Broadband Photodetector Based on Organolead Halide Perovskite. *Adv. Funct. Mater.* **24**, 7373–7380 (2014).
4. Dou, L. *et al.* Solution-processed hybrid perovskite photodetectors with high detectivity. *Nat. Commun.* **5**, 5404 (2014).
5. Horváth, E. *et al*. Nanowires of methylammonium lead iodide ($CH_3NH_3PbI_3$) prepared by low temperature solution-mediated crystallization. *Nano Lett.* **14**, 6761−6766 (2014).
6. Guo, Y. *et al*. Air-stable and solution-processable perovskite photodetectors for solar-blind UV and Visible light. *J. Phys. Chem. Lett.* **6**, 535−539 (2015).
7. Lee, Y. *et al*. High-performance perovskite–graphene hybrid photodetector. *Adv. Mater.* **27**, 41–46 (2015).
8. Dong, R. *et al*. High-gain and low-driving-voltage photodetectors based on organolead triiodide perovskites. *Adv. Mater*. **27**, 1912–1918 (2015).
9. Lin, Q. *et al*. Low noise, IR-blind organohalide perovskite photodiodes for visible light detection and imaging. *Adv. Mater*. **27**, 2060–2064 (2015).
10. Fang, Y. *et al.* Resolving weak light of sub-picowatt per square centimeter by hybrid perovskite photodetectors enabled by noise reduction. *Adv. Mater.* **27**, 2804–2810 (2015).
11. Li, D. *et al.* High performance organic-inorganic perovskite-optocoupler based on low-voltage and fast response perovskite compound photodetector. *Sci. Rep.* **5**, 7902 (2015).
12. Deng, H. *et al.* Growth, patterning and alignment of organolead iodide perovskite nanowires for optoelectronic devices. *Nanoscale* **7**, 4163–4170 (2015).
